# Case report: Diffuse hyperplastic perilobar nephroblastomatosis complicated by a unilateral Wilms tumour: diagnosis, treatment and follow-up

**DOI:** 10.1186/s13104-018-3502-7

**Published:** 2018-06-19

**Authors:** Bruce Gao, Emeka Nzekwu, Anthony Jonathan Cook, Shelley Jane Spaner

**Affiliations:** 1 The University of Calgary, Cumming School of Medicine, 3330 Hospital Dr NW, Calgary, AB T2N 4N1 Canada; 2 Department of Radiology, The University of Calgary, 3330 Hospital Dr NW, Calgary, AB T2N 4N1 Canada; 3 Department of Surgery (Urology), The University of Calgary, 3330 Hospital Dr NW, Calgary, AB T2N 4N1 Canada

**Keywords:** Kidney, Cancer, Oncology, Paediatrics, Wilms, Tumor, Nephroblastomatosis, Surgery, Radiology, Urology

## Abstract

**Background:**

Nephroblastomatosis is an uncommon pathologic process characterized by the presence of persistent embryonic nephrogenic rests. Progression to Wilms tumour occurs in an estimated 35% of patients. Cure rates are based on histologic findings and disease stage and have improved from 10% in the 1920s to over 90% today.

**Case presentation:**

We report a case of a 9-month-old female presenting with a 2-month history of abdominal distension. Ultrasonographic and computed tomographic assessments demonstrated features consistent with bilateral, diffuse, hyperplastic perilobar nephroblastomatosis (DHPLNB) for which she underwent chemotherapy. Magnetic resonance imaging 6 weeks following commencement of chemotherapy revealed a mass concerning for unilateral Wilms tumor for which she underwent partial nephrectomy. Pathology confirmed DHPLNB with a unilateral Wilms tumor.

**Conclusion:**

3.5 year radiographic follow up demonstrates complete recovery. To our knowledge, there are no similar cases with imaging depiction recently published. With potential for malignant transformation into Wilms tumour and low survival rate for late diagnosed Wilms tumors, it is important to recognize nephroblastomatosis early, both clinically and radiographically to improve overall patient prognosis.

## Background

Nephroblastomatosis is an uncommon pathology, with distinct but rare radiological findings. With potential for malignant transformation into Wilms tumour and low survival rate for late diagnosed Wilms tumors, it is important to recognize nephroblastomatosis early, both clinically and radiographically to improve overall patient prognosis. The following describes a case of Wilms tumour complicating bilateral nephroblastomatosis.

## Case presentation

### History and physical

A 9-month-old female presented with a 2-month history of abdominal distension and weight loss.

The patient underwent an uncomplicated birth following an unremarkable pregnancy at 39-weeks gestational age via elective caesarean section. The toddler attained normal developmental milestones aside from the inability to roll prone from supine. Initially at the 70th percentile at birth for weight, she presented at the 30th percentile with stable height and head circumference (50th percentile). The patient did not exhibit any clinical features of Beckwith Wiedemann Syndrome including omphalocele, macroglossia and macrosomia.

There is no consanguinity, no family history of recurrent malignancies, haematological or renal conditions.

### Investigations

Initial limited ultrasonographic study of the abdomen demonstrated massively enlarged kidneys with loss of corticomedullary differentiation. The right kidney measured 13.1 cm and the left measured 15 cm (normal approximately 6 cm) [1]. Multiple ill-defined hypoechoic areas were seen randomly interspersed within the renal parenchyma bilaterally suggesting presence of nephrogenic rests and therefore nephroblastomatosis. It was not possible to rule out the presence of Wilms tumour within the nephrogenic rests on ultrasound. No evidence of hydronephrosis, hydroureter or free fluid was seen.

**Fig. 1:**
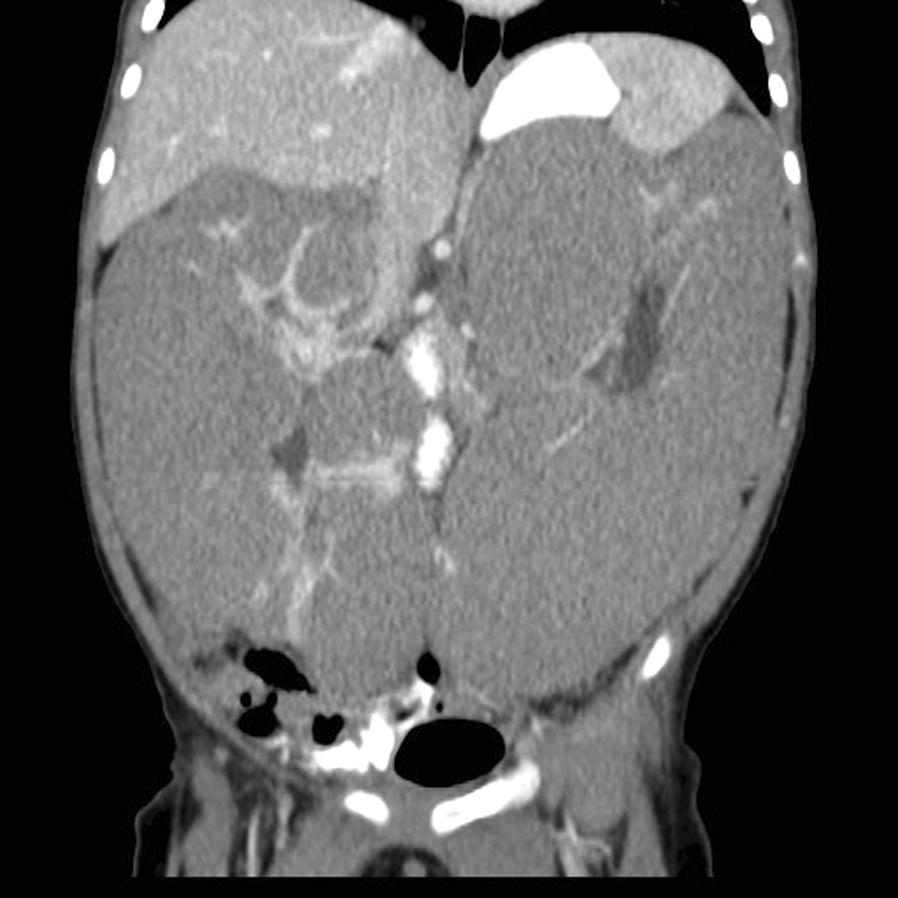
Coronal slice of CT abdomen/pelvis with IV and oral contrast showing bilateral homogenous, diffuse renal enlargement with low-attenuating perinephric nephrogenic rests

Computed tomography (CT) of the abdomen and pelvis with IV and oral contrast demonstrated homogenous, diffuse, renal enlargement and loss of normal architecture with renal parenchyma replaced by homogenous low attenuating peripheral masses bilaterally (Fig. 1). There were two ill-defined hypodense areas in the medial aspect of the left kidney concerning for malignancy. Residual normal renal parenchyma was present as areas of striate enhancement, hyperdense in comparison to the thick rind of peripheral nodules. Based on clinical presentation and CT findings, the patient was diagnosed with bilateral DHPLNB.

**Fig. 2:**
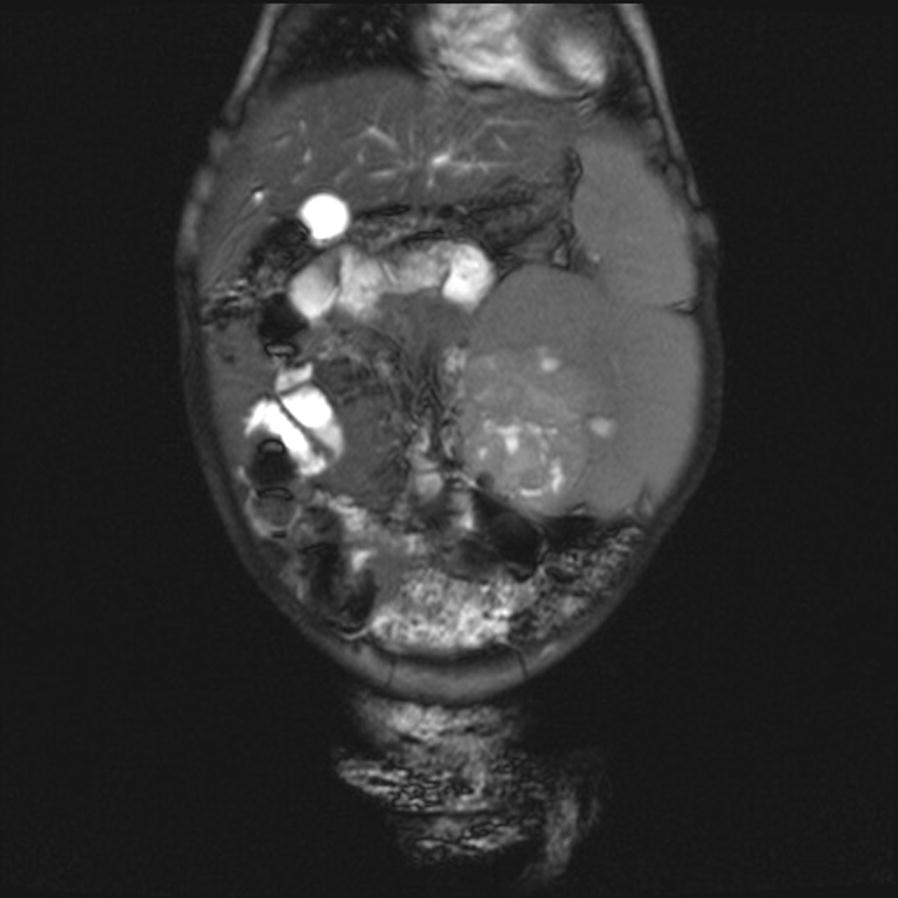
Coronal slice of T1 gadolinium enhanced MRI performed after 6 weeks of chemotherapy revealing a 6 × 6.3 cm heterogeneous enhancing lesion in the medial aspect of the left kidney with multiple linear and round tiny cysts. A dominant gadolinium enhancing 1 cm area to the left of this heterogeneous mass is suspicious for complicating Wilms tumor

Gadolinium enhanced magnetic resonance imaging (MRI) of the abdomen performed after 6 weeks of chemotherapy demonstrated hypointense peripheral masses on T1/T2 images with thick septations suggestive of bilateral DHPLNB. A small heterogeneously enhancing lesion with multiple small linear and round cysts in the medial left kidney was characterized on T1 imaging, suspicious for a superimposed Wilms tumor (Fig. 2). Diffusion weighted imaging was not conducted.

No metastases were seen on CT chest with contrast. Pathology after left partial nephrectomy confirmed Wilms tumor.

Cytogenetic analysis was conducted revealing a normal female karyotype (46XX). No other tumour markers or genetic testing was performed.

### Differential diagnosis

- Bilateral DHPLNB complicated by a left renal mass (likely Wilms Tumour)
- Renal lymphoma (uncommon in infants and young children).

### Treatment/intervention

The patient was treated with COG protocol AREN 0534 EE-4A chemotherapy, including vincristine and dactinomycin for 20 weeks. Due to the presence of the superimposed left renal mass, a left partial nephrectomy and perihilar lymph node dissection was performed. Following the surgery, she also received left flank radiotherapy to a dose of 1080 cGy in 6 fractions.

### Outcome and follow-up

Pathology was consistent with DHPLNB and resection of a Wilms tumor. Resection margins were positive. Nodal dissection was negative. Following surgical resection and chemotherapy, the patient was followed with MRI every 3 months for a year. Thereafter, the patient was followed with MRI every 6 months for 2 years. Currently, the patient is now on surveillance with ultrasounds every 3–4 months.

## Discussion and conclusions

Nephroblastomatosis is a premalignant condition typified by multiple residual embryonal cells known as nephrogenic rests [2]. Nephrogenic rests are clusters of embryonic metanephric cells found incidentally in 1% of infants that can exist as single or multiple, focal or diffuse lesions within the renal parenchyma. They are further classified based on their location within the kidney as perilobar or intralobar [3]. Perilobar rests are restricted to the peripheral renal cortex and are associated with fetal overgrowth disorders [4] while intralobar rests occur randomly throughout the renal parenchyma as focal lesions [5, 6]. Hyperplastic perilobar nephroblastomatosis is generally bilateral and the renal cortical surface consists partially or entirely of hyperplastic nephroblastic tissue in DHPLN [7]. The disease is also associated with an increased risk of developing Wilms tumour [5].

Sonographic features of nephroblastomatosis are typified by enlarged diffusely hypoechoic kidneys. CT imaging demonstrates low-attenuating poorly enhancing peripheral nodularity with scattered patches of adjacent normally enhancing renal parenchyma. On MRI, this corresponds to peripheral nodules with low-signal intensity on T1 and T2-weighted sequences when compared to normal renal parenchyma [8].

In contrast to the multiplicity of the subcapsular nephrogenic rests which define nephroblastomatosis and frequently serve as precursors to nephroblastomas (Wilms tumours); Wilms tumours typically manifests as solid intrarenal masses distorting the renal parenchyma and collecting system [9]. They are known to metastasize by direct extension displacing adjacent structures however they do not elevate or encase the aorta which differentiates this pathology from neuroblastomas [9]. They demonstrate heterogenous contrast enhancement on CT and MRI and are intrinsically of low signal intensity on T1-weighted imaging and high signal intensity on T2-weighted imaging [9]. Quantitative MRI techniques such as diffusion-weighted imaging (DWI) have proven valuable in differentiating intracranial tumours, but no such role has been validated for differentiation of nephroblastomatosis from Wilms tumour. However, preliminary data suggest that tissue cellularity is an important determinant of apparent diffusion coefficient which may help in terms of early prediction of Wilms tumour therapy response [10].

Treatment of nephroblastomatosis complicated by Wilms tumor incorporates multimodal therapy including surgery, chemotherapy and radiation, with more aggressive initial therapeutic protocols in the presence of poor prognostic factors. Poor prognostic factors include anaplastic tumour histology, tumour staging, unfavourable molecular and genetic markers and patient age greater than 2 years old [11]. Fortunately for this child, tumour histology was not anaplastic and there was no metastatic disease. In addition, one of the main reasons for this favorable outcome was the ability of the radiologist to identify the tumour while the child was still young. Overall, Wilms tumour 5-year survival rates have increased from 20% in the 1960s to 90% in 2005 clinical trials [11].

Gadolinium-enhanced T1 and T2-weighted MR imaging is often used to monitor regression or progression of nephrogenic tissue. Tumor regression is evident when there is a reduction in size of the nephrogenic tissue as well as decreased enhancement of the original lesion reflecting increased fibrosis and devascularization of the nephrogenic rests [12]. Increased lesion dimensions and signal intensity on T2-weighted images raises suspicion for tumor progression [12]. DHPLN has an unpredictable clinical course, with lesions varying in size over time. There is an increased risk of renal insufficiency, prolonged hypertension, and pulmonary complications if untreated. Additionally, as described in this case, there is an increased risk of development of a Wilms tumor.

## Learning points

- Nephroblastomatosis is an uncommon pathology characterized by the presence of persistent embryonic nephrogenic rests
- Wilms tumour complicates approximately 35% of cases
- Diagnostic imaging can be useful for the diagnosis of diffuse hyperplastic perilobar nephroblastomatosis, and screening for complications such as Wilms tumour.

## Abbreviations

DHPLNB: diffuse hyperplastic perilobar nephroblastomatosis
CT: computed tomography
MRI: magnetic resonance imaging
DWI: diffusion-weighted imaging
